# The impact of prepregnancy overweight and obesity on newborn glucose-lipid metabolism: An observational study

**DOI:** 10.1097/MD.0000000000042440

**Published:** 2025-06-13

**Authors:** Xia Chen, Jianmin Zhang, Huanhuan Li, Yuanru Tang, Yan Zhang, Ziwen Ma, Yifan Hu

**Affiliations:** a Department of Gynecology and General Practice, Huangpu District Bund Community Health Service Center, Shanghai, China; b Department of Gynecology and Obstetrics, Pudong New Area Health Care Hospital for Women and Child Gynecological Clinic, Shanghai, China.

**Keywords:** glycolipid metabolism, newborn, obesity, overweight, pregnancy

## Abstract

This study seeks to explore the association between maternal prepregnancy excessive weight and metabolic changes in glucose and lipids during early gestation, with a particular focus on its implications for neonatal metabolic health. The results aim to support the development of evidence-based strategies that enhance maternal metabolic health before conception, ultimately promoting better neonatal outcomes. This study included 1467 women of reproductive age who were registered in a community in Huangpu District, Shanghai, between 2017 and 2021. Based on their prepregnancy body mass index, participants were classified into an overweight/obese group and a normal-weight group. The study compared neonatal metabolic indicators from offspring delivered by the 2 maternal groups, including triglycerides (TG), total cholesterol, high-density lipoprotein cholesterol (HDL-C), low-density lipoprotein cholesterol (LDL-C), fasting blood glucose, leptin, fasting insulin, and the insulin resistance index, to evaluate the potential impact of maternal prepregnancy weight status on neonatal metabolic health. Newborns delivered by mothers in the overweight/obese group exhibited significantly higher levels of leptin, triglycerides (TG), total cholesterol, low-density lipoprotein cholesterol (LDL-C), fasting insulin, fasting blood glucose, and insulin resistance index. In comparison, levels of high-density lipoprotein cholesterol (HDL-C) were significantly lower compared to those born to mothers in the normal-weight group (*P* < .05). Preconception overweight and obesity are closely associated with neonatal glucose and lipid metabolism. Implementing effective weight management strategies before pregnancy is essential for optimizing neonatal metabolic health.

## 1. Introduction

Over the past 2 decades, the prevalence of overweight and obesity among women of reproductive age has increased significantly, contributing to a growing public health concern regarding maternal and neonatal metabolic health.^[1]^ A multi-center national survey conducted in 2022 found that the overweight rate among Chinese adults was 34.29%, and the obesity rate was 11.24%.^[2]^ Prepregnancy overweight and obesity can profoundly alter maternal glucose and lipid metabolism, increasing the risk of metabolic complications during pregnancy.^[3]^ These alterations can extend beyond the maternal system, affecting fetal metabolic programming and neonatal metabolic health.^[4–6]^

The developmental origins of health and disease (DOHaD) hypothesis suggests that the intrauterine metabolic environment plays a critical role in shaping offspring metabolic health.^[7]^ Prepregnancy obesity is associated with elevated maternal lipid levels, insulin resistance, and systemic inflammation, which can cross the placenta and disrupt fetal metabolic homeostasis.^[6]^ Studies have demonstrated that neonates born to overweight or obese mothers exhibit higher birth weight, increased fat mass, and altered lipid profiles, predisposing them to an increased risk of metabolic disorders later in life.^[8]^

Despite growing recognition of these risks, the specific mechanisms through which maternal prepregnancy overweight, and obesity impact glucose and lipid metabolism in offspring remain inadequately understood. This study aims to investigate the effects of prepregnancy overweight and obesity on neonatal glucose and lipid metabolism, providing new insights into the metabolic interplay between mother and fetus.

## 2. Research methods

### 2.1. Study population

**Figure 1.**
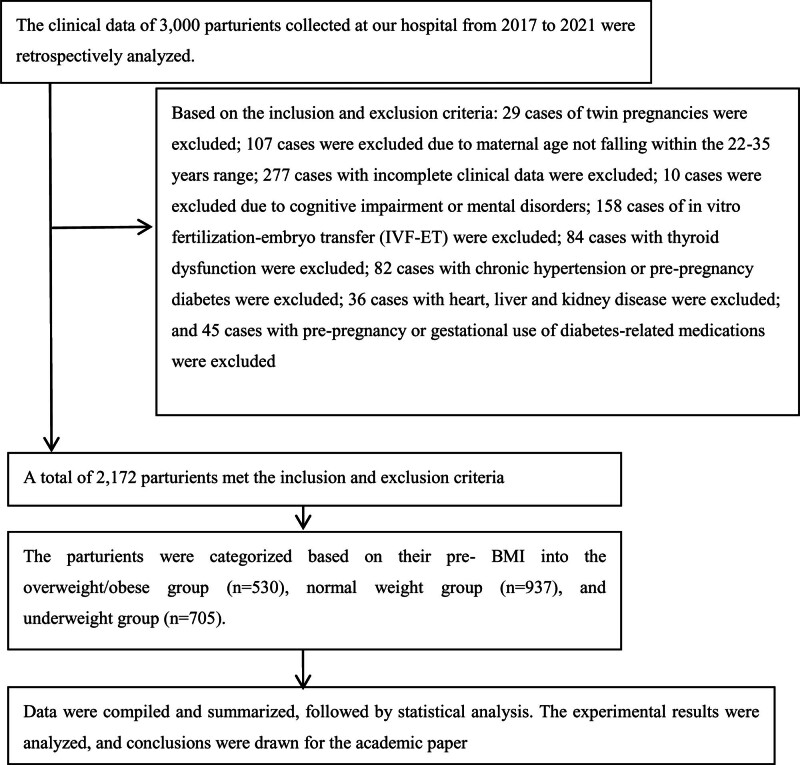
A flow diagram of the study participants.

This retrospective study analyzed 1467 pregnant women from 2017 to 2021. The inclusion criteria were: singleton natural conception; visible primitive heart tube pulsation detected in early pregnancy ultrasound; absence of prepregnancy comorbidities influencing the study; maternal age between 22 and 35 years; complete clinical records. Exclusion criteria: severe insufficiency of vital organs such as heart, liver, and kidneys; cognitive impairment affecting participation; loss to follow-up; fetal abnormalities; prepregnancy diabetes or chronic hypertension. The participant screening procedure and results flowchart are presented in Figure 1. All participants gave their informed consent, and the research received approval from the Ethics Committee. Study Subgroups: Participants were categorized into 2 groups based on their body mass index (BMI): an overweight/obese group (BMI ≥ 24.0 kg/m^2^, n = 530) and a normal-weight group (BMI 18.5–24.0 kg/m^2^, n = 937).

### 2.2. Observation indicators

Neonatal metabolic markers include leptin, TG, TC, HDL-C, LDL-C, fasting insulin, fasting blood glucose (FBG), and insulin resistance index (IRI).

### 2.3. Statistical analysis

Data analysis was performed using SPSS 26.0 (Chicago). Categorical variables were compared using the *χ*^2^ test, while continuous variables were analyzed using *t*-tests. To explore the relationship between BMI and metabolic markers, Spearman rank correlation was utilized. Statistical significance was set at a *P*-value of < .05.

## 3. Results

### 3.1. Comparative analysis of maternal characteristics by prepregnancy BMI categories

In the overweight and obese group, the mean maternal age was 29.15 ± 3.03 years, with educational levels distributed as follows: 36 women had junior high school education or lower, 78 had completed high school, and 416 had at least a college-level education. The group included 107 primiparous women. In the normal-weight group, the mean maternal age was 28.82 ± 3.15 years, with 98 women having junior high school education or lower, 98 with a high school education, and 741 with a college education or higher. There were 191 primiparous women in this group. Statistical analysis revealed no significant differences between the 2 groups in terms of age, educational level, and primiparity (*P* > .05) (Table 1).

**Table 1:** Comparison of basic data between the overweight/obese group and the normal-weight group.

Group	Age (year)			Degree of education			Primipara
	<30	≥30	Average age	Below junior high school	High school and secondary school	Junior college degree or above	
Overweight and obesity group (530)	418 (78.87)	112 (21.13)	29.15 ± 3.03	36 (6.79)	78 (14.72)	416 (78.49)	107 (20.19)
Normal-weight group (937)	758 (80.90)	182 (19.42)	28.82 ± 3.15	98 (10.46)	98 (10.46)	741 (79.08)	191 (20.38)
*χ*^2^*/t* value	0.593		2.133	3.028			0.203
*P*-value	.441		.119	.082			.652

Data are presented as mean ± standard deviation or frequency and percentage.

### 3.2. Comparative analysis of leptin and glucose-lipid indicators in newborns from overweight obese and normal-weight groups

In the comparative analysis, newborns from the overweight and obese group exhibited significantly higher levels of leptin, TG, TC, LDL-C, fasting insulin, and FBG, along with a higher IRI, compared to their counterparts in the normal-weight group. In contrast, HDL-C levels were significantly lower in the overweight and obese group (*P* < .05) (Table 2).

**Table 2:** Comparative analysis of leptin and glucose-lipid indicators in newborns from overweight/obese and normal-weight groups.

Group	Leptin (ng/mL)	TG (mmol/L)	TC (mmol/L)	LDL-C (mmol/L)	HDL-C (mmol/L)	Fasting insulin (mU/L)	FBG (mmol/L)	IRI
Overweight and obesity group (530)	1.25 ± 0.39	0.69 ± 0.17	1.42 ± 0.31	0.84 ± 0.15	1.34 ± 0.18	20.03 ± 4.70	22.09 ± 4.00	19.8 ± 6.32
Normal-weight group (937)	0.67 ± 0.14	0.41 ± 0.18	1.23 ± 0.39	0.57 ± 0.13	1.80 ± 0.34	13.35 ± 3.46	11.79 ± 2.99	9.03 ± 2.54
*t* value	7.183	6.57	2.282	7.648	7.632	6.282	11.344	8.168
*P*-value	<.001	<.001	0.025	<.001	<.001	<.001	<.001	<.001

Except for leptin, blood glucose, and lipid levels are xˉ±s.

FBG = fasting blood glucose, HDL-C = high-density lipoprotein cholesterol, IRI = insulin resistance index, LDL-C = low-density lipoprotein cholesterol, TC = total cholesterol, TG = triglycerides.

### 3.3. Analysis of the correlation between prepregnancy BMI and newborn leptin, IRI, and levels of blood glucose and lipid

Spearman’s rank correlation analysis revealed a positive correlation between prepregnancy BMI and the levels of leptin, TG, TC, HDL-C, LDL-C, fasting insulin, FBG levels, and IRI (*R* = 0.652, 0.571, 0.327, 0.715, 0.584, 0.459, 0.794, 0.795 *P* < .05) (Table 3).

**Table 3:** Correlation analysis of prepregnancy BMI and newborn leptin and blood glucose-lipid levels.

Spearman rank correlation coefficient correlation		Leptin	TG	TC	HDL-C	LDL-C	Fasting insulin	FBG	IRI
Prepregnancy body mass index	Correlation coefficient	.652*	.571*	.327*	.715*	.584*	.459*	.794*	.795*
	Significance (2-tailed)	<.001	<.001	<.001	<.001	<.001	<.001	<.001	<.001

BMI = body mass index, FBG = fasting blood glucose, HDL-C = high-density lipoprotein cholesterol, IRI = insulin resistance index, LDL-C = low-density lipoprotein cholesterol, TC = total cholesterol, TG = triglycerides.

* a statistically significant correlation at the 0.01 level (2-tailed).

## 4. Discussion

In recent years, as national living standards continue to rise and dietary patterns evolve, the incidence of overweight and obesity has been on the rise, posing significant challenges to both physical and mental health.^[9,10]^ Existing studies have shown that prepregnancy overweight and obesity significantly increase the physical and psychological health risks for both mothers and infants. These metabolic abnormalities are not only associated with various health risks for newborns but may also lead to short-term and long-term complications, increasing the likelihood of developing cardiovascular diseases later in life,^[11]^ as well as potentially causing immune dysfunction and other health issues.^[12]^ According to an August 2020 directive from the Beijing Municipal Health Commission, strengthening nutrition services and management during prepregnancy and pregnancy is particularly emphasized. Therefore, this study analyzes the impact of prepregnancy overweight and obesity on the glucose-lipid metabolic indicators in newborns in a Shanghai community. Furthermore, it examines the correlation between prepregnancy overweight/obesity and newborn glucose-lipid metabolism. This study provides a scientifically substantiated and rational foundation for prenatal healthcare interventions targeting overweight and obese women in the community. By implementing targeted interventions, this study seeks to enhance glucose-lipid metabolism in newborns.

The findings of the present study indicate that, compared with the normal-weight cohort, the overweight and obese cohort exhibited elevated levels of newborn leptin, triglycerides, total cholesterol (TC), LDL cholesterol, fasting insulin, FBG, and IRI. Conversely, HDL cholesterol levels were found to be lower in the overweight and obese cohort. All variables show varying degrees of correlation, with a negative correlation with HDL-C (*R* = 0.715) and positive correlations of different magnitudes with TG, TC, LDL-C, FBG, and IRI (*R* = 0.652, 0.571, 0.327, 0.584, 0.457, 0.794, 0.795). The findings indicate that maternal overweight and obesity before pregnancy may exert an influence on the glucose-lipid metabolic processes in newborns. Extensive research from both animal experiments and clinical studies^[13,14]^ has consistently indicated that excessive weight before pregnancy, coupled with nutritional excess during pregnancy, exerts detrimental effects on offspring. These adverse impacts manifest as a heightened risk of various conditions in the progeny, including but not limited to obesity, insulin resistance, diabetes mellitus, and atherosclerosis. Current research predominantly links the adverse effects of maternal overweight and obesity on offspring’s glucose-lipid metabolism to the increased birth weight of infants born to mothers in these categories.^[15]^ This elevation in birth weight is believed to disrupt endocrine functions, potentially altering metabolic pathways.^[16,17]^ Overnutrition in mothers with obesity not only predisposes the fetus to excessive growth but also adversely influences the development of the fetal hypothalamic appetite control center. This interaction potentially disrupts the fetal energy balance system through various pathways including leptin secretion, insulin secretion, and adipocyte metabolism, thereby escalating the risk of macrosomia and neonatal metabolic disorders, particularly those related to glucose-lipid metabolism.^[18]^ In addition, the increased adipose tissue in macrosomic infants elevates the risk of diabetes and obesity in later life.^[19]^ From a genetic analysis perspective, over 500 genomic loci associated with obesity traits have been identified, confirming that both obesity and its related clinical features are heritable and constitute major risk factors for diseases in progeny.^[20]^ The study by Luís et al^[21]^ suggests that overweight and obese mothers may influence the metabolic health of their progeny by altering gene expression, which, due to its hereditary effects, could increase the risk of metabolic disorders such as diabetes in adulthood. Hence, in terms of macro-causality, maternal prepregnancy overweight and obesity increase the risk of macrosomia and newborn disorders of glycolipid metabolism, and controlling prepregnancy body mass is clinically important in preventing disorders of glycolipid metabolism in the progeny.

Prepregnancy overweight and obesity have a significant impact on fetal organ development. A study by Salmeri et al^[22]^ indicates that abnormal prepregnancy weight (either underweight or overweight) is an independent risk factor for congenital heart defects. Being overweight before pregnancy increases the risk of congenital heart defects through metabolic and hormonal changes. The relationship between prepregnancy weight and different types and severities of defects varies, with higher risks observed, particularly in cases of obesity. Therefore, managing prepregnancy weight is crucial for reducing the risk of congenital heart defects in the fetus. Although this study did not explore this aspect, it will be an important direction for future research.

Although this study provides an in-depth evaluation of neonatal metabolic disorders using biochemical markers and gene expression profiles, its retrospective single-center nature imposes inherent limitations. As a result, the reliability of its data may be lower compared to large-scale, multi-center prospective randomized controlled studies. Moreover, prepregnancy BMI classification relied on self-reported weight at the first prenatal visit, which introduces potential bias. Future studies should adopt a multi-center approach, employ prospective study designs, and utilize objectively validated BMI measurements to enhance accuracy. Given Huangpu District’s economic prosperity, socioeconomic disparities may affect maternal and neonatal metabolic parameters, necessitating careful interpretation. For instance, higher income levels, enhanced nutritional access, and superior healthcare services may elevate maternal lipid and glucose levels, distinguishing this population from those in resource-limited settings. Thus, economic and geographic factors may introduce variability in the study findings, limiting their applicability to maternal populations in lower-income regions. Future research should incorporate geographically and socioeconomically diverse cohorts, employ matched sampling strategies, and adjust for potential confounding variables. Furthermore, integrating machine-learning-based predictive models that combine multiple biochemical and genetic indicators may offer superior diagnostic and prognostic value. Expanding biomarker panels to include inflammatory cytokines, endocrine hormones, and novel metabolic indicators could further improve predictive sensitivity and specificity.

## 5. Conclusion

In conclusion, prepregnancy overweight is significantly associated with metabolic imbalances in both mothers and newborns, particularly in glucose and lipid regulation. Prepregnancy overweight status may lead to early disruptions in maternal-fetal metabolic balance, which in turn affects neonatal metabolic parameters such as glucose, lipids, and leptin levels. Therefore, weight control strategies for overweight and obese women before conception may improve offspring metabolic profiles, reducing the likelihood of metabolic dysfunction in the offspring. To achieve this, a multidisciplinary team, including nutritionists, obstetricians, and endocrinologists, should be integrated into preconception healthcare services, focusing on structured weight management programs for overweight women. Healthcare providers should collaborate with obstetric institutions to emphasize personalized dietary and lifestyle interventions to optimize prepregnancy BMI, ensuring that weight management strategies are both practical and sustainable for long-term metabolic health benefits.

## Acknowledgments

We would like to acknowledge everyone for their helpful contributions to this paper.

## Author contributions

**Conceptualization:** Xia Chen, Jianmin Zhang.

**Data curation:** Xia Chen.

**Formal analysis:** Jianmin Zhang, Huanhuan Li.

**Methodology:** Ziwen Ma, Yifan Hu.

**Visualization:** Yuanru Tang, Yan Zhang.

**Writing – original draft:** Xia Chen.

**Writing – review & editing:** Xia Chen, Jianmin Zhang, Huanhuan Li.
